# Combination of pioglitazone and clomiphene citrate versus clomiphene citrate alone for infertile women with the polycystic ovarian syndrome

**DOI:** 10.1186/s12905-021-01448-5

**Published:** 2021-08-17

**Authors:** Maliheh Amirian, Sedigheh Shariat Moghani, Faezeh Jafarian, Masoumeh Mirteimouri, Shima Nikdoust, Shabnam Niroumand, Maryam Salehi, Aryan Payrovnaziri

**Affiliations:** 1 Department of Obstetrics and Gynecology, Fellowship of Infertility, School of Medicine, Infertility Center of Mashhad, University of Medical Sciences, Mashhad, Iran; 2 Department of Midwifery, Nursing and Midwifery Care Research Center, Mashhad University of Medical Sciences, Mashhad, Iran; 3 North Khorasan University of Medical Sciences, Shirvan, Iran; 4 Department of Obstetrics and Gynecology, Omoalbanin Hospital, School of Medicine, Mashhad University of Medical Sciences, Mashhad, Iran; 5 Mashhad University of Medical Sciences, Mashhad, Iran; 6 School of Medicine, Mashhad University of Medical Sciences, Mashhad, Iran; 7 GREENWICH University England, London, England

**Keywords:** PCOS, Infertility, Pioglitazone, Ovulation induction

## Abstract

**Background:**

Anovulation is one of the common causes of infertility. Polycystic ovary syndrome (PCOS) is the most common disorder with chronic Anovulation. To the best of our knowledge, insulin resistance relates significantly to PCOS. Therefore administration of insulin-sensitizing drugs such as pioglitazone can be used for ovulation stimulation in PCO patients.

**Methods:**

After obtaining approval from the Ethics Committee of Mashhad University of Medical Sciences, 61 patients with PCOS were enrolled in the study based on inclusion/ exclusion criteria. Patients were divided into two groups. The first group received 30 mg (mg) of pioglitazone daily from the second day of the menstrual period. The second one received a placebo. 150 mg clomiphene citrate was administered from the third to the seventh day of the menstrual cycle. Vaginal sonography was performed in all women, and in cases with the mature follicle, intrauterine insemination was conducted after human chorionic gonadotropin injection. Ovary stimulation and pregnancy rate were compared between groups.

**Results:**

There were no differences between groups regard to demographic characteristics and infertility type. Body mass index was higher in the pioglitazone group (28.3 ± 3.8 versus 26.2 ± 3.5, *P* value = 0.047). The size of the follicle was not significantly different between groups (2.2 ± 1.4 versus 1.3 ± 1.1, *P* value = 0.742). pregnancy rate [4 (12.9%) versus 4 (13.3%), *P* value = 1] had no differences between groups.

**Conclusion:**

Although the number of follicles was higher in the pioglitazone group, our study showed no differences in ovary stimulation and pregnancy rate.

## Background

Infertility affects about 10–15% of couples. 30% of the causes of infertility in women are due to ovulation failure [1]. Polycystic ovary syndrome (PCOS) is the most apparent and common disorder associated with chronic ovulation failure [2]. PCOS prevalence is about 15–20% when the European Society for Human Reproduction and Embryology and the American Society for Reproductive Medicine (ESHRE/ASRM) diagnostic criteria were used [3].

Abnormal levels of lipoproteins are typical in patients with PCOS with an increase in total cholesterol (Chol), triglycerides (TG), low-density lipoproteins (LDL), and high levels of high-density lipoprotein (HDL) and apoptotic A-I [4–6]. According to a report, the most significant change in lipid is the reduction of HDL. Hyperinsulinemia and insulin resistance (IR) are common in PCOS. Mostafa et al. found that about 46% of Egyptian PCOS women had IR [4, 7]. Insulin disrupts steroidogenesis in the ovary independently from gonadotropin secretion I PCOS [1]. Insulin receptors and insulin-like growth factor-1 (IGF-I) exist in ovarian stromal cells [5]. Reduction of autophosphorylation, a specific disorder related to insulin receptor-mediated signal sending, was detected in 50% of women with PCOS [3].

Abnormal glucose metabolism may significantly improve weight loss; weight loss may reduce hyperandrogenism and reinstate ovulation function [7]. Obese women with insulin resistance, caloric restriction, and weight loss reduce the severity of insulin resistance. On the other hand, a decrease in insulin concentrations reduces androgen production [8].

Nowadays, clomiphene citrate is the recommended treatment for inducing ovulation in women with PCOS. The association of insulin resistance is significantly observed with polycystic ovary syndrome, so the use of drugs that increase the sensitivity of the insulin receptor, such as metformin and β-thiazolidinedione, has been considered in the treatment of these patients. Treatment of insulin resistance might induce ovulation, especially in obese women with higher insulin resistance degrees [9].

Insulin resistance means decreased glucose response to insulin with subsequent hyperinsulinemia, which leads to elevated triglyceride, and decreased HDL-cholesterol, glucose intolerance, and cardiovascular risks [10]. Pioglitazone, used as a treatment for type 2 diabetes, directly affects peripheral insulin sensitivity. In some recent studies, pioglitazone has been shown to reduce intra-ovarian stromal blood flow. It may help improve ovarian stimulation and In vitro fertilization (IVF) outcomes in PCOS patients. Coffler showed that pioglitazone could induce ovulation significantly in patients with hyperinsulinemia [11].

So far, no study has examined the effect of pioglitazone on the fertility of our patients. So, we hypothesized that pioglitazone as an insulin sterilizer agent might improve the ovulation rate and pregnancy in PCOS patients. This study aimed to use pioglitazone for a successful pregnancy, including chemical and clinical pregnancy and the number of medium and large-size follicles in infertile women with PCOS.

## Methods

Mashhad University of medical sciences supervised this randomized clinical trial study from 2014 until 2017, and 61 PCOS patients who were referred to the Milad Infertility Center for the treatment of infertility were enrolled using a non-probabilistic sampling method. The Ethical Committee of Mashhad University of Medical Sciences approved the hold on "March 15, 2014" and written informed consent was obtained from all participants.

### Patients' selection

Inclusion criteria were infertile women aged 18–38 years who had normal hysterosalpingography and normal spermogram. The diagnosis of the polycystic ovarian syndrome is based on the AES criteria (Androgen Excess Society 2006), based on the above criteria: (1) Hirsutism or hyperandrogenic symptoms. (2) Ovarian dysfunction as oligomenorrhea, or ultrasonographic criteria for polycystic ovaries as neck lace-like appearance; and (3) rollouts of secondary causes, such as ovarian and adrenal tumor and pituitary adenoma. If the menstrual cycle was oligomenorrhea, or the number of peripheral follicles 2–9 millimeters of the ovary was more significant than nine on the Ferriman-Gallway score, the patient was diagnosed with polycystic ovary syndrome.

Patients with a history of chronic cardiovascular disease, chronic kidney disease, diabetes, thyroid disease, and pulmonary disease were excluded.

After selecting eligible patients, they were divided into two groups by simple random sampling using computer software. The envelope method was used for random allocation of patients in study groups. In this way, random numbers will be placed in sealed envelopes. The contents of the envelope were not visible from the outside. In group A, each pack contained 30 tablets of pioglitazone, 30 mg, and 15 tablets of clomiphene, and in group B, 30 placebo tablets and 15 clomiphene tablets were placed. Patients were blinded to the assigned treatment.

All patients underwent vaginal sonography on the second day of menstruation day and enrolled in the study if they had no ovarian cysts over 20 mm.

The number of medium size follicles and large size follicles and the endometrium thickness were assessed on the tenth or eleventh day of menstruation. The chemical and clinical pregnancy rates were evaluated.

### Intervention

The first group received 30 mg pioglitazone daily; from the second day of menstruation, the second group received a placebo. 150 mg clomiphene citrate was administered to both groups between the third and seventh days of the menstrual cycle. Vaginal sonography was obtained on day 10 or 11. Human chorionic gonadotropin (HCG) and then intrauterine insemination injection (IUI) was considered in women with endometrial thickness more than 7 mm and follicle larger than 16mm.

In cases with 5 days of delayed menstrual period, a blood sample was performed for evaluating the βHCG level. Pioglitazone-related side effects and the number of follicles more massive than 16 mm, and endometrial thickness were assessed during the study period. Eventually, ovary stimulation and pregnancy rate were compared between groups.

### Sample size and statistical analyses

The sample size was calculated using PASS 11 software and comparing the average number of follicles in each group. By default, the first type error was 5%, and the second type error was 20%. We estimated 22 patients in each group, but due to the probable loss of patients, 30 participants were considered in each group.

Data were entered in SPSS version 16. Initially, the characteristics of each group were described by descriptive statistical methods, including mean and standard deviation for continuous variables and number plus frequency for categorical variables. Then, to compare the quantitative variables in two study groups, after assessing the condition of normality with the Kolmogorov–Smirnov test, an independent *t*-test or Mann–Whitney-U test was used. Qualitative variables were compared using the chi-square test. In all statistics, *P*-value less than 0.05 was considered as the significance level.

## Results

**Fig. 1:**
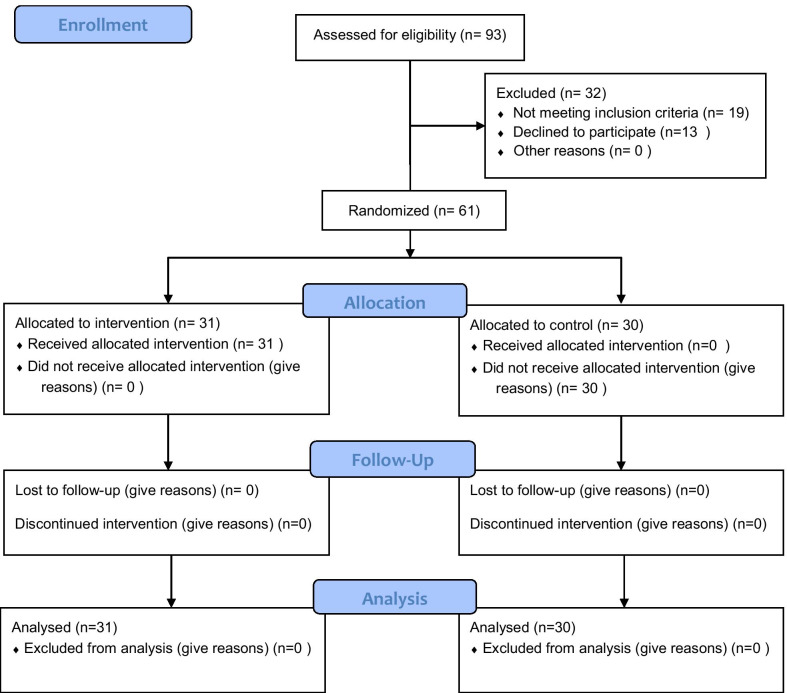
Consort diagram. This Consort Diagram illustrates the process

Concerning inclusion criteria, 93 females were enrolled in the study, 19 had exclusion criteria, and 13 gave up. Thirty patients were classified as the placebo group, and the other 31 were the intervention one. The CONSORT algorithm was presented in Fig. 1. The demographic characteristics of women are shown in Table 1. There were no differences between groups regard to demographic characteristics and infertility type. The mean age was 28.20 ± 5.46 in the intervention group and 27.07 ± 4.18 in the control group, and this difference was not statistically significant. However, body mass index (BMI) was higher in the pioglitazone group.

**Table 1:** Demographic characteristics of patients

Characteristics		All patients (n = 61)	Intervention (n = 31)	Placebo (n = 30)	*P* value
Age (mean ± SD)		27.6 ± 4.8	28.2 ± 5.4	27.0 ± 4.1	0.383
Infertility type (N/%)	Primary	48 (85.7)	24 (82.8)	24 (88.9)	0.707
	Secondary	14.3 (8)	17.2 (5)	11.1 (3)	
Infertility duration ( mean ± SD)		5.6 ± 3.1	5.8 ± 3.8	5.4 ± 2.5	0.551
BMI ( mean ± SD)		27.3 ± 3.8	28.3 ± 3.8	26.2 ± 3.5	0.047

Patient sonography findings such as the number of medium size follicles, the number of large follicles, the maximum size of the follicle, and the thickness of the endometrium are summarized in Table 2. As it shows, the follicles' size was not significantly different between groups except for medium size follicles.

**Table 2:** Size of follicles and endometrial thickness in two study groups

	Intervention (n = 31)	Placebo (n = 30)	*P* value
Medium size follicles	3.8 ± 2.5	1.3 ± 0.9	0.037
Large size follicles	2.2 ± 1.4	1.3 ± 1.1	0.742
Maximum size of the follicle	16.6 ± 2.7	17.5 ± 2.6	0.055
The thickness of the endometrium	7.6 ± 2.9	7.6 ± 2.2	0.952

Medium size: defined as 11–15 cm. Large size: defined as 16–24 cm

Information on the results of the treatment of ovulation stimulation, such as the amount of ovulation per cycle, chemical and clinical pregnancy rate, were shown in Table 3. Ovary stimulation and pregnancy rate had no differences between groups.

**Table 3:** The results of the treatment of ovulation stimulation

	In all patients (n = 61)	In intervention group (n = 31)	In placebo group (n = 30)	*P*-value
Amount of ovulation per cycle (N/%)	42 (89)	18 (58.1)	24 (80)	0.742
Chemical pregnancy rate (N/%)	10 (16.4)	4 (12.9)	6 (20)	0.508
Clinical pregnancy rate (N/%)	8 (13.1)	4 (12.9)	4 (13.3)	1

## Discussion

The results of this study demonstrated that there was a significant difference in the number of ovulation stimulation in patients receiving pioglitazone. Ultrasonography was performed on the 10th day of menstruation and showed that the mean number of follicles was significantly higher in the intervention group. Our findings confirmed the result of a study in 2012 about the role of pioglitazone in ovulation stimulation in PCOS patients with hyperinsulinemia [12]. Morley et al. also reported an increase in ovulation after pioglitazone administration in PCOS patients [13].

Ovulation and pregnancy rates did not differ between the two study groups. This may be due to the duration of pioglitazone used before starting clomiphene. Ota demonstrated that in 2008, the results showed that of the nine patients who took pioglitazone for 12–30 weeks before clomiphene citrate, seven cases became pregnant [14]. Kim in 2010 showed that the number of follicles was significantly lower after pioglitazone administration. Also, in his study, clinical pregnancy was higher in the pioglitazone group, but this difference was not statistically significant. This finding was against our results but can be explained by the patient's selection criteria (including clomiphene-resistant patients) [15].

Ota showed that pioglitazone could improve the pregnancy rate in PCOS patients resistant to clomiphene and dexamethasone [14]. It seems selecting PCOS cases with hyperandrogenism should be performed more carefully. Patients in the Ota project had different hormonal levels, which can affect the result of pioglitazone therapy. Hormonal levels did not significantly differ before and after interventions in our study.

There was no significant difference in the number of large follicles and endometrial thickness between intervention and control groups in our study. However, the number of medium-sized follicles was significantly higher in the intervention group.

In the present study, BMI was higher in the intervention group, which means hyperinsulinemia might be more probable in this group and influence the outcome, although this difference was not statistically significant between the two groups.

None of our patients experienced side effects. Liver function tests did not statistically significant change during the study period.

Our study's main limitation was the study's design as a case–control project, which led to a BMI difference between the two groups. So, the result might be influenced by this difference. However, no similar study with this two-drug regimen was performed on patients in our area. However, due to the effect of pioglitazone on insulin resistance, it seems that if the patient is on pioglitazone treatment for a more extended period before starting the clomiphene diet, the success rate will increase. So, more studies are suggested to determine the best duration of pioglitazone use.

## Conclusion

Although the number of follicles was higher in the pioglitazone group, our study showed no differences in ovary stimulation and pregnancy rate between groups.

Indeed, we were effectively treating the specific problems of infertility, bleeding due to uterine dysfunction, and hirsutism in the past. Now we have the opportunity (and indeed the duty) to provide interventions that can prevent or rectify some of the infertility's metabolic complications (which significantly impact overall health and quality and quantity of life).

## Data Availability

All tables and the study results were provided based on the study raw data and it is available. SPSS file will be sent to you upon your request.

## Abbreviations

PCOS: Polycystic ovary syndrome
IUI: Intrauterine insemination
HCG: Human chorionic gonadotropin
BMI: Body mass index
TSH: Thyroid-stimulating hormone
PRL: Prolactin
FSH: Follicle-stimulating hormone
LH: Luteinizing hormone
LFT: Liver function test
BUN: Blood urea nitrogen
Cr: Creatine
CBC: Complete blood count
FBS: Fasting blood sugar

## References

[1] Kini S (2012). Polycystic ovary syndrome: diagnosis and management of related infertility. Obstet Gynaecol Reprod Med.

[2] McCartney CR, Marshall JC (2016). Polycystic ovary syndrome. New Engl J Med.

[3] Abdelazim IA, Kanshaiym S (2019). Abdelazim and Sakiye endocrinopathy associated with polycystic ovary syndrome: case reports. J Fam Med Prim Care.

[4] Toulis KA, Goulis DG, Kolibianakis EM, Venetis CA, Tarlatzis BC, Papadimas I (2009). Risk of gestational diabetes mellitus in women with polycystic ovary syndrome: a systematic review and a meta-analysis. Fertil Steril.

[5] Pall M, Azziz R, Beires J, Pignatelli D (2010). The phenotype of hirsute women: a comparison of polycystic ovary syndrome and 21-hydroxylase–deficient nonclassic adrenal hyperplasia. Fertil Steril.

[6] Moran LJ, Pasquali R, Teede HJ, Hoeger KM, Norman RJ (2009). Treatment of obesity in polycystic ovary syndrome: a position statement of the Androgen Excess and Polycystic Ovary Syndrome Society. Fertil Steril.

[7] Mostafa R, Al-Sherbeeny MM, Abdelazim IA, Elshehawy Y, Wahba KA, Abuel-Fadle A (2015). Frequency of insulin resistance in Egyptian women with polycystic ovary syndrome. MOJ Womens Health.

[8] Xu Y, Wu Y, Huang Q (2017). Comparison of the effect between pioglitazone and metformin in treating patients with PCOS: a meta-analysis. Arch Gynecol Obstet.

[9] Morishita M, Endo T, Baba T, Kuno Y, Ikeda K, Kiya T (2018). pioglitazone is effective for multiple phenotypes of the Zuckerfa/fa rat with polycystic ovary morphology and insulin resistance. J Ovarian Res.

[10] Mostafa R, Al-Sherbeeny MM, Abdelazim IA, Elshehawy Y, Wahba KA, Abuel-Fadle A (2015). Frequency of insulin resistance in Egyptian women with polycystic ovary syndrome. MOJ Women's Health.

[11] Gupta A, Jakubowicz D, Nestler JE (2016). Pioglitazone therapy increases insulin-stimulated release of d-chiro-inositol-containing inositolphosphoglycan mediator in women with polycystic ovary syndrome. Metab Syndr Relat Disord.

[12] Sangeeta S (2012). Metformin and pioglitazone in polycystic ovarian syndrome: a comparative study. J Obstet Gynaecol India.

[13] Morley LC, Tang T, Yasmin E, Norman RJ, Balen AH (2017). Insulin-sensitizing drugs (metformin, rosiglitazone, pioglitazone, d-chiro-inositol) for women with polycystic ovary syndrome, oligo amenorrhoea, and subfertility. Cochrane Database Syst Rev.

[14] Ota H, Goto T, Yoshioka T, Ohyama N (2008). Successful pregnancies treated with pioglitazone in infertile patients with polycystic ovary syndrome. Fertil Steril.

[15] Kim CH, Jeon GH, Kim SR, Kim SH, Chae HD, Kang BM (2010). Effects of Pioglitazone on ovarian stromal blood flow, ovarian stimulation, and in vitro fertilization outcome in patients with polycystic ovary syndrome. Fertil Steril.

